# Hepatoprotective effects of *Curcuma* *xanthorrhiza* Roxb. extract via free radical scavenger, inhibiting apoptosis and inflammation mechanisms in acetaminophen-induced liver injury

**DOI:** 10.22038/ijbms.2025.82500.17833

**Published:** 2025

**Authors:** I Nyoman Ehrich Lister, Linda Chiuman, Maya Sari Mutia, Hartono Hartono, Ermi Girsang, Annisa Firdaus Sutendi, Hanna Sari Widya Kusuma, Dhanar Septyawan Hadiprasetyo, Wahyu Widowati

**Affiliations:** 1 Faculty of Medicine, Universitas Prima Indonesia, Medan 20118, North Sumatera, Indonesia; 2 Biomolecular and Biomedical Research Center, Aretha Medika Utama, Bandung 40163, Indonesia; 3 Faculty of Pharmacy, Jenderal Achmad Yani University, Cimahi 40531, Indonesia; 4 Faculty of Medicine, Maranatha Christian University, Bandung 40164, West Java, Indonesia

**Keywords:** Acetaminophen Antioxidants, Apoptosis, Curcuma xanthorrhiza, Free radical scavengers Liver injury

## Abstract

**Objective(s):**

Acetaminophen (APAP)-mediated liver injury poses a significant public health concern. *Curcuma xanthorrhiza* extract (CXE) has been traditionally used for its hepatoprotective properties. This research aimed to assess the hepatoprotective effects of CXE in APAP-mediated hepatotoxicity by investigating the modulatory effects of CXE on key biomarkers, including Interleukin (IL), namely, (IL-6), IL-10, IL-1β, Nitric Oxide (NO), Lactate Dehydrogenase (LDH), and the genes expression related to apoptosis-like Caspase-3 (Casp-3), Casp-9, and genes related to liver metabolic c-Jun N-terminal Kinase (JNK), in APAP-mediated HepG2 cells.

**Materials and Methods:**

APAP-induced HepG2 cells were treated with different concentrations of CXE. IL-6, IL-10, IL were measured using an Enzyme-linked Immunosorbent Assay (ELISA) and NO, LDH were measured using colorimetric assay. Gene expression was analyzed using quantitative Real-Time Reverse Transcription (qRT-PCR).

**Results:**

CXE significantly reduced IL-1β and IL-6 levels, enhanced IL-10 production, and attenuated NO levels in APAP-mediated hepatotoxicity. CXE also suppressed the expression of Casp-9, Casp-3, JNK, and LDH levels. The study presented a concentration-dependent response, with 125 μg/ml CXE exhibiting the most pronounced effects. CXE effectively modulated immune responses, decreased oxidative stress, and inhibited apoptotic and inflammatory pathways in APAP-mediated hepatotoxic cells.

**Conclusion:**

These studies highlight the CXE potential as a therapeutic candidate for liver disorders, particularly in drug-mediated liver injury.

## Introduction

The liver, a vital organ responsible for metabolism and detoxification, is susceptible to harm from drugs, environmental toxins, and other foreign substances (1). Acetaminophen (APAP), widely used as an antipyretic and analgesic, is generally safe at the recommended dosage but could cause severe liver toxicity in overdose, potentially leading to liver failure (2, 3). APAP-mediated liver injury has emerged as a significant concern.

APAP is metabolized via sulfation and glucuronidation, leading to non-toxic metabolites excreted through urine (4, 5). However, hepatic cytochrome P450 enzymes transform APAP into N-Acetyl-P-benzo Quinone Imine (NAPQI), a toxic intermediate that depletes glutathione and causes hepatocyte necrosis (6, 7). The accumulation of NAPQI triggers oxidative stress, ATP depletion, and mitochondrial damage, leading to necrotic cell death (8).

An imbalance of cytokines, namely Interleukin-1β (IL-1β), IL-6, and IL-10, can lead to liver diseases, including fibrosis and acute inflammation (9). Reactive oxygen species (ROS), particularly NO, activate the c-Jun N-terminal Kinase (JNK) phosphatase and Mitogen-activated Protein Kinase Phosphatase- 1 (MKP-1), which regulate JNK, a key player in apoptosis and liver inflammation (10, 11). Activation of Caspase-9 (Casp-9) initiates a cascade leading to programmed cell death via Casp-3 and Casp-7 (12). Elevated Lactate dehydrogenase (LDH) was also found in acute liver damage, acting as a pro-inflammatory agent (13). Targeting these pathways offers a promising strategy for mitigating liver injury (14). 

Due to the side effects of synthetic hepatoprotective drugs, there is increasing interest in herbal medicine, which has been used historically for liver disorders (15, 16). Among these, *Curcuma xanthorrhiza* Roxb. extract (CXE) is widely recognized for its hepatoprotective and antioxidant properties (17). Traditional medicine suggests its efficacy against liver disorders, diabetes, cancer, and hypertension (18). CXE exhibits various bioactivities, namely antioxidant, anti-inflammatory, hepatoprotective, and antimicrobial (19).

 Thus, this study aims to assess the hepatoprotective effects of CXE by evaluating its role in reducing inflammation, oxidative stress, and apoptosis through the modulation of NO, cytokines (IL-Iβ, IL-10, and IL-6), LDH, and the expression of apoptosis-related genes (Casp-3 and Casp-9) and liver stress-related gene JNK.

## Materials and Methods

### Preparation of CXE

*C. xanthorrhiza* extract utilized in this research was processed at Fathonah Amanah Siddiq Tabligh (FAST) located in Depok, West Java, Indonesia, bearing Certificate of Analysis (CoA) No. Batch 00110201069. The extraction process is based on Good Manufacturing Practices (GMP). The rhizome of *C. xanthorrhiza* was extracted using a 70% ethanol solvent. The resulting extract was then mixed with lactose to produce dry extract powder (20, 21).

### Quality testing of CXE

The quality of CXE was assessed according to the regulation of Indonesia Food and Drug Authority, number 32 of 2019, which encompassed organoleptic testing, physical characterization, and microbiological contamination assessment.

### Cell culture and APAP-mediated HepG2 cells

HepG2 cell (human hepatocellular carcinoma) was acquired from Aretha Medika Utama, Indonesia (ATCC, HB-8065™). The cells were cultured in complete media consisting of Modified Eagle Medium (MEM) (Biowest, L0416-500), 10% fetal bovine serum (FBS) (Biowest, S1810), 1% nanomycopulitine (Biowest, LX16), and 1% antibiotic-antimycotic (Gibco, 15240062). Liver toxicity was mediated using a 40 mM concentration of APAP (Sigma Aldrich, A7085). The cells were then washed with PBS after reaching 80-90% cell density, and trypsin-EDTA (Gibco, 25200072) was utilized to detach the cells. 5x10^5^ cells were seeded into six-well plates and then incubated for 24 hr with 5% CO_2_ at 37 ^°^C. After the CXE was administered into the cells, it was cultured for 24 hr. The experimental groups were categorized as follows: I) Negative control (Normal cells); II) Positive control (40 mM of APAP); III) Vehicle control (Positive control+DMSO 1%); IV) 40 mM of APAP+CXE 5 μg/ml; V) 40 mM of APAP+CXE 25 μg/ml; VI) 40 mM of APAP+CXE 125 μg/ml. Following exposure, the cell samples were centrifuged for 10 min at 1600 rpm, and the ELISA assay was performed on the collected supernatant (22-24).

### Quantification of IL-1β, IL-10, and IL-6

Interleukin levels (IL-Iβ, IL-10, and IL-6) were measured using the Human ELISA kit (Elabscience, E-EL-H0149; E-EL-H6156; E-EL-H6154) consecutively. Using a spectrophotometer multiscan GO (Thermo Scientific, 1510-00778C), the absorbance was determined at 450 nm, following the instructions provided in the manufacturer’s kit manual (25, 26). 

### Nitric oxide (NO) assay

The sample, as much as 10 µl, was added to a mixture of PBS and 40 µl 10 mM sodium nitroprusside (Merck, 106541) before the incubating process for two hours at room temperature. The Griess reagent was prepared by mixing 2% H_3_PO_4_ (Merck, 100573), 1% sulfanilamide (Merck, 222799), and 0.1% N-(1-naphtyl) ethylenediamine dihydrochloride (Sigma Aldrich, 222488). The reagent was administered (100 µl) into the 96-well microplate, and the NO level was quantified at 546 nm using a microplate reader (20, 27).

### Lactate dehydrogenase (LDH) assay

The kit from Elabscience (E-BC-K046-M) was utilized to evaluate the LDH activity, following the manufacturer’s guidelines. The cell culture secretion was collected, and the LDH contained in the secretion medium was quantified. This enabled the evaluation of the toxicity mediated by CXE (28, 29).

### Expression of Casp-9, Casp-3, and JNK gene

The expression of Caspases (Casp-3 and Casp-9) and JNK was evaluated using quantitative qRT-PCR Agilent. RNA isolation was carried out following the instructions provided by the Direct-zol RNA Kit (Zymo, R2073). The total RNA was measured by quantifying the absorbance at 260/280 nm with a spectrophotometer, Multiscan GO. cDNA was obtained using the SensiFAST cDNA Synthesis Kit (Meridian Bioscience, BIO-65054). The primer sequence (Macrogen) is shown in Table 1. The qPCR conditions comprised an initial pre-denaturation at 95 ^°^C for five minutes, denaturation at 95 ˚C for 5 min, 40 thermal cycles of 94 ^°^C for 50 sec, 40 cycles of 58 ^°^C for 50 sec, and 72 ^°^C for 50 sec, with a final extension was performed at 72 ^°^C (30).

## Results

### CXE quality

The quality of CXE was determined by conducting organoleptic, physical characteristics, and microbiological contamination tests. The results indicate compliance with the standards of Indonesian Food and Drug Authority No. 32 of 2019 in relation to the standards for traditional medicines safety and quality, as shown in Table 2.

### CXE effect towards IL-1β, IL-10, and IL-6 level in APAP-induced HepG2 cells

**Figure 1:**
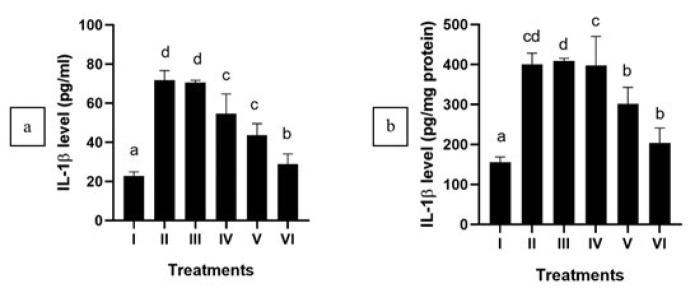
Effect of various concentrations of CXE on IL-1β level in APAP-mediated HepG2 cells

**Figure 2:**
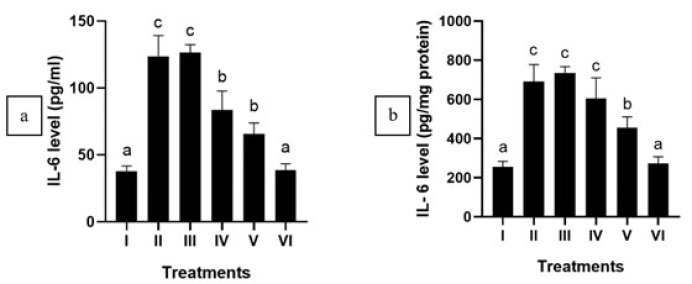
Effect of various concentrations of CXE on IL-6 levels in APAP-induced HepG2 cells

**Figure 3:**
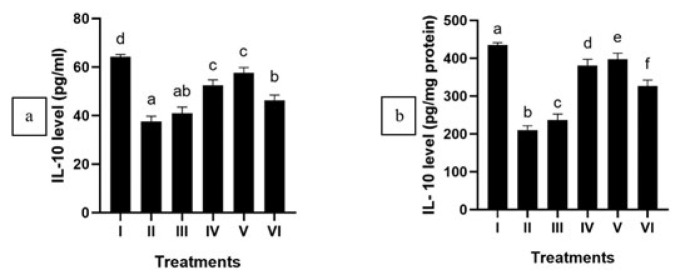
Effect of various concentrations of CXE on IL-10 level in APAP-induced HepG2 cells

APAP administration significantly increased IL-6 and IL-1β levels and down-regulated IL-10 within APAP-mediated HepG2 cells (*P*<0.05). After the treatment of CXE, a notable reduction in IL-6 and IL-1β levels was detected (*P*<0.05) (Figures 1 and 2), while IL-10 shows a significant increase in APAP-mediated HepG2 cells (Figure 3). This outcome suggests that CXE holds the potential to inhibit pro-inflammatory cytokines production in APAP-induced HepG2 cells and regulate the anti-inflammatory cytokines, as evidenced by the significant difference observed in contrast with the positive control group. The concentration of CXE at 125 μg/ml exhibited the highest efficacy for treating the APAP-mediated HepG2 cells.

### CXE effect towards NO and LDH level in APAP-induced HepG2 cells

**Figure 4:**
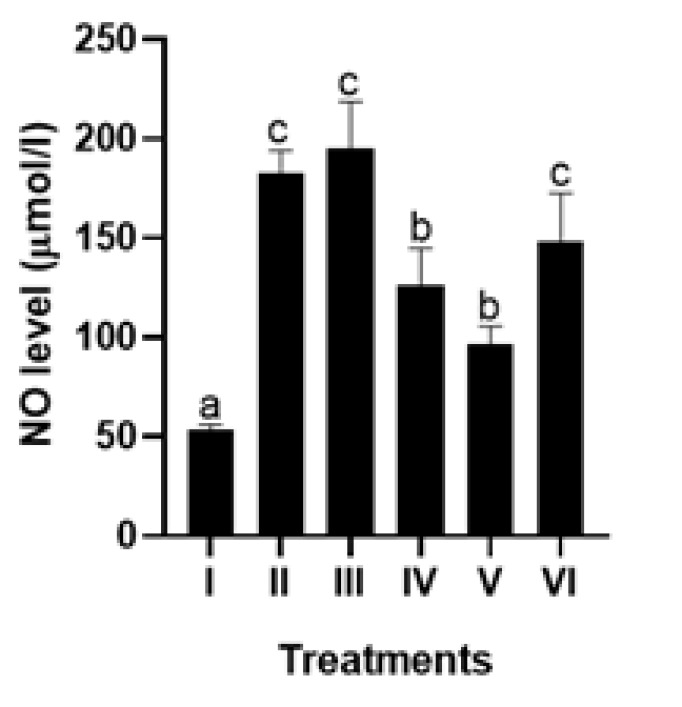
Effect of various concentrations of CXE on NO level in APAP-mediated HepG2 cells

**Figure 5:**
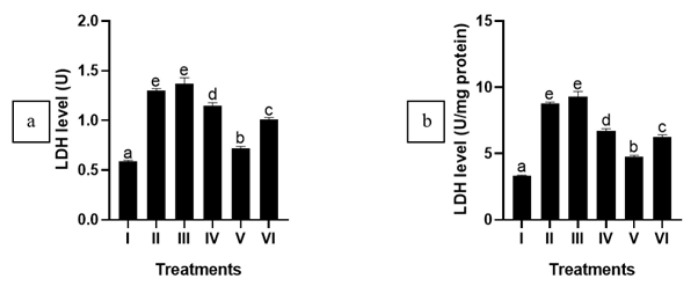
CXE treatment effect on LDH level in APAP-induced HepG2 cells

APAP induction increased NO and LDH levels in HepG2 cells significantly (*P*<0.05), which notably decreased significantly when HepG2 cells subjected to injury were treated with 5 μg/ml and 25 μg/ml CXE (*P*<0.05)(Figures 4 and 5). This data indicates that CXE can potentially reduce NO and LDH levels in the liver injury model. 

### CXE effect toward Casp-3, Casp-9, and JNK gene expression in APAP-induced HepG2 cells

**Figure 6:**
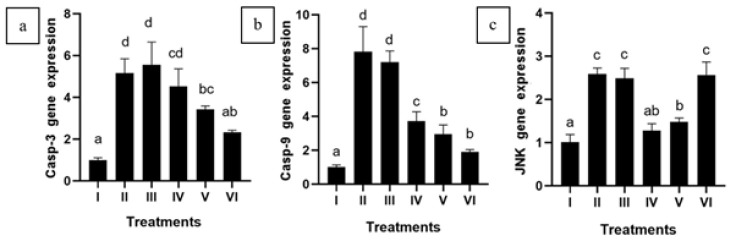
Effect of various concentrations of CXE on Casp-3, Casp-9, and JNK gene expression in APAP-induced HepG2 cells

Casp-3, Casp-9, and JNK gene expression noticeably increased (*P*<0.05) in HepG2 cells mediated by APAP. Treatments with CXE showed a substantial decrease (*P*<0.05) in Casp-3 and Casp-9 gene expression compared to the group of APAP-mediated HepG2 cells. The expression of JNK was also found to be lower in diseased HepG2 cells that had been treated with CXE (Figure 6).

## Discussion

*C. xanthorrhiza, *commonly known as Javanese turmeric, has long been used as an alternative medicine for liver diseases due to its rich bioactive compounds, including xanthorrhizol, curcuminoids, flavonoids, and terpenoids (31,32). Among these, xanthorrhizol is recognized as the key hepatoprotective compound, demonstrating superior activity compared to curcumin (33, 34). The hepatoprotective potential of *C. xanthorrhiza* is attributed to its antioxidant properties, which scavenge free radicals and reduce oxidative damage (31). The presence of flavonoids and phenolic compounds in *C. xanthorrhiza* allows it to effectively neutralize ROS such as H_2_O_2_, NO, and DPPH, thus preventing oxidative stress-related liver damage (20). 

NO and LDH serve as essential biomarkers for assessing the hepatoprotective effects of natural compounds. NO, a signaling molecule, is pivotal in regulating liver function (35). LDH is an enzyme released from damaged liver cells and is known as a marker for liver damage (36). NO was discovered to be elevated in patients with liver cirrhosis and plays an important role as a vasodilator of hepatic vascular nature (37). Various studies have investigated the hepatoprotective effects of CXE, demonstrating its potential therapeutic benefits (17, 34, 38). Treatment with 5 μg/ml and 25 μg/ml CXE significantly reduces NO and LDH production. This suggests CXE could safeguard liver cells by mitigating oxidative stress and inflammation.

In this study, APAP-induced hepatotoxicity elevated pro-inflammatory cytokines, namely IL-6 and IL-1β, while decreasing IL-10, a key anti-inflammatory cytokine (39, 40). Treatment with CXE modulated cytokine expression by reducing IL-6 and IL-1β levels while restoring IL-10, indicating its anti-inflammatory effects. Inflammatory diseases are characterized by an excessively active immune response, often resulting in tissue damage and chronic inflammation (41). In this study, CXE modulates several cytokines synthesis, namely IL-1β, IL-6, and IL-10, regulating the immune response and mitigating inflammation.

APAP exposure significantly up-regulated the expression of apoptotic markers Casp-3, Casp-9, and JNK, which are critical mediators of apoptosis and inflammation in liver cells (42, 43). Our results showed that CXE treatment markedly decreased the expression of these apoptotic markers, supporting its role in hepatoprotection. This is consistent with studies reporting that curcumin reduces Casp-3 and Casp-9 levels in liver disease models (44). This suggests that CXE may protect liver cells by inhibiting the apoptotic and inflammatory pathways triggered by APAP-mediated liver injury and inflammation.

**Figure 7:**
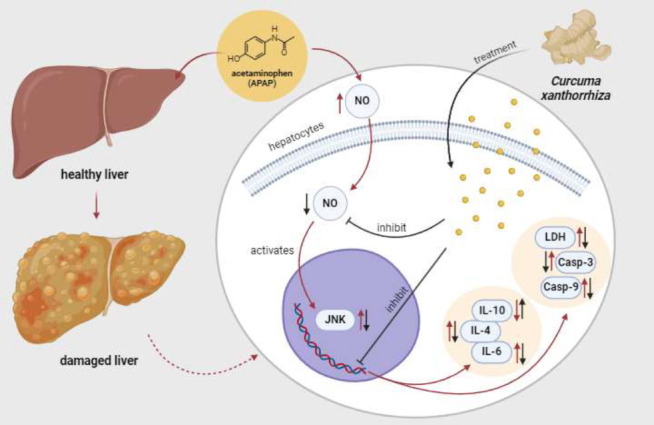
Proposed mechanism of APAP-induced HepG2 cells treated with* Curcuma xanthorrhiza*

CXE exhibits strong hepatoprotective effects by modulating inflammatory and apoptotic pathways. The synergistic effects of the various bioactive compounds in CXE, combined with the higher concentration, contribute to enhanced hepatoprotective effects. The mechanism of CXE as hepatoprotective in damaged HepG2 cells is described in Figure 7. Sustainable APAP consumption leads to liver damage, as indicated by increased NO levels and activation of JNK, IL-6, IL-1β, LDH, and apoptotic markers (Casp-9 and Casp-3). Treatment with CXE exerts its hepatoprotective effects by modulating these key pathways. CXE reduces NO levels, effectively inhibiting JNK activation and preventing the downstream effects of oxidative stress. This inhibition suppressed the apoptotic markers, including Casp-9 and Casp-3, thereby preventing excessive hepatocyte death. Furthermore, CXE modulates the inflammatory response by decreasing IL-6 and IL-1β levels while increasing IL-10 production. IL-10 is an anti-inflammatory cytokine that regulates immune homeostasis. The reduction in LDH levels further suggests that CXE preserves cell integrity and prevents extensive liver damage.

**Table 1:** Primer sequence design of target gene in APAP-induced HepG2 cells

Gen	Primer Sequence (5' - 3')	Product length (bp)	Annealing (^°^C)	Cycle	Reference
GADPH human	F: GCCAAAAGGGTCATCATCTC	178	58	40	NM_001357943.2
	R: TGAGTCCTTCCACGATACCA				
Casp-3 human	F: AGAACTGGACTGTGGCATTGAG	191	58	40	NM_001354783.2
	R: GCTTGTCGGCATACTGTTTCAG				
Casp-9 human	F: CATGCTCAGGATGTAAGCCA	116	58	40	NM_001229.5
	R: AGGTTCTCAGACCGGAAACA				
JNK human	F: GTCGTTGCATCTGTTTCTCCA	129	58	40	NM_001278547.2
	R: CACCAAGAAGCCTGACAG				

*Data were obtained from the NCBI entrez nucleotide database

**Table 2:** Quality characteristics of *Curcuma xanthorrhiza* extract (CXE)

Item	Specification	Method	Result
Organoleptic			
Form	Fine powder	Sensory analysis Sensory analysis Sensory analysis Sensory analysis	Qualified
Color	Yellow		Qualified
Odor	Typical curcuma odor		Qualified
Flavor	Bitter		Qualified
Physical characteristic			
Extract ratio	1:1	-	Qualified
80 mesh testing	≥ 90%	Testing sieve	Qualified
Solubility	Low solubility in water	-	Qualified
Water content	< 10%	432/01/2019/QC	Qualified
Microbiological contamination /1 g			
Total plate count (TPC)	≤10^5^ colony	415/03/2019/QC	2.1 x 10^2^
Yeast cell count	≤10^3^ colony	415/03/2019/QC	1.1 x 10^1^
*E. Coli*	≤10 colony	415/03/2019/QC	< 1.0 x 10^1^
*Enterobacteriaceae*	≤10^3^ colony	415/03/2019/QC	1.0 x 10^2^
*Clostridia*	Negative	415/03/2019/QC	Negative
*Salmonella*	Negative	415/03/2019/QC	Negative
*Shigella*	Negative	415/03/2019/QC	Negative

(a) IL-1β level (pg/ml) and (b) IL-1β level (pg/mg protein) in hepatotoxic cells model. * The data represented as mean±standard deviation (SD). I) Negative control (normal cells); II) Positive control (APAP-mediated cells); III) Positive control+DMSO 1% (vehicle control); IV) Positive control+CXE 5 μg/ml; V) Positive control+CXE 25 μg/ml; VI) Positive control+CXE 125 μg/ml. The letters (a, b, c, and d) for Figure 1a and (a, b, c, cd, and d) for Figure 1b present significant differences between various concentrations of CXE toward IL-1β level (Tukey HSD *post hoc *test, *P<*0.05)

CXE: *Curcuma Xanthorrhiza* extract, APAP: Acetaminophen

(a) IL-6 level (pg/ml) and (b) IL-6 level (pg/mg protein) in hepatotoxic cells model. * The data represented as mean±SD. I) Negative control (normal cells); II) Positive control (APAP-mediated cells); III) Positive control+DMSO 1% (vehicle control); IV) Positive control+CXE 5 μg/ml; V) Positive control+CXE 25 μg/ml; VI) Positive control+CXE 125 μg/ml. The letters (a, b, and c) present significant differences between various concentrations of CXE toward the IL-6 level (Tukey HSD *post hoc* test, *P<*0.05)

CXE: *Curcuma Xanthorrhiza* extract, APAP: Acetaminophen

(a) IL-10 level (pg/ml) and (b) IL-10 level (pg/mg protein) in hepatotoxic cells model. * The data represented as mean±SD. I) Negative control (normal cells); II) Positive control (APAP-mediated cells); III) Positive control+DMSO 1% (vehicle control); IV) Positive control+CXE 5 μg/ml; V) Positive control+CXE 25 μg/ml; VI) Positive control+CXE 125 μg/ml. The letters (a, ab, b, c, and d) for Figure 3a and (a, b, c, d, e, and f) for Figure 3b show significant differences between various concentrations of CXE toward IL-10 levels (Tukey HSD *post hoc* test, *P<*0.05)

CXE: *Curcuma Xanthorrhiza* extract, APAP: Acetaminophen

* The data represented as mean±SD. I) Negative control (normal cells); II) Positive control (APAP-mediated cells); III) Positive control+DMSO 1% (vehicle control); IV) Positive control+CXE 5 μg/ml; V) Positive control+CXE 25 μg/ml; VI) Positive control+CXE 125 μg/ml. The letters (a, b, and c) present significant differences between various concentrations of CXE toward NO level (Tukey HSD *post hoc* test, *P<*0.05)

CXE: *Curcuma Xanthorrhiza* extract, APAP: Acetaminophen

(a) LDH level (U) and (b) LDH level (U/mg protein) in hepatotoxicity cells model. * The data represented as mean±SD. I) Negative control (normal cells); II) Positive control (APAP-mediated cells); III) Positive control+DMSO 1% (vehicle control); IV) Positive control+CXE 5 μg/ml; V) Positive control+CXE 25 μg/ml; VI) Positive control+CXE 125 μg/ml. The letters (a, b, c, d, and e) present significant differences between various concentrations of CXE toward LDH level (Tukey HSD *post hoc* test, *P<*0.05)

CXE: *Curcuma Xanthorrhiza* extract, APAP: Acetaminophen

(a) Casp-3, (b) Casp-9, and (c) JNK gene expression in APAP-mediated HepG2 Cells. * The data represented as mean±SD. I) Negative control (normal cells); II) Positive control (APAP-mediated cells); III) Positive control+DMSO 1% (vehicle control); IV) Positive control+CXE 5 μg/ml; V) Positive control+CXE 25 μg/ml; VI) Positive control+CXE 125 μg/ml. The letter (a, ab, bc, cd, and d) for Figure 6a, (a, b, c, and d) for Figure 6b, and (a, ab, b, and c) for Figure 6c present significant differences between various concentrations of CXE toward Casp-3, Casp-9, and JNK gene expression (Tukey *post hoc *test, *P<*0.05)

CXE: *Curcuma Xanthorrhiza *extract, APAP: Acetaminophen

APAP induction increased NO levels and activated JNK, IL-6, IL-1β, LDH, Casp-9, and Casp-3. CXE administration reduced NO levels, inhibited JNK activation, and decreased IL-6 and IL-1β levels, while IL-10 production increased, LDH levels reduced, and Casp-9 and Casp-3 gene expression decreased

CXE: *Curcuma Xanthorrhiza *extract, APAP: Acetaminophen

## Conclusion

This research demonstrates the potential clinical application of CXE as a hepatoprotective therapy for patients with liver damage caused by medication. CXE is proven to suppress NO, pro-inflammatory (IL-6, IL-1β, and LDH), and apoptosis genes (Casp-9, Casp-3, and JNK) in APAP-mediated liver cells while increasing the expression of an anti-inflammatory agent (IL-10), which can be a potent target for liver disease treatment. Human clinical trials are necessary to confirm these findings and establish the most effective dosage and treatment duration. Further research should explore the molecular mechanisms of CXE and its synergistic effects with other herbal remedies or conventional drugs. Long-term safety evaluation is essential to ensure the extended use of CXE.

## Declaration

We have not used any AI tools or technologies to prepare this manuscript.

## References

[1] Beier JI, Arteel GE (2021). Environmental exposure as a risk-modifying factor in liver diseases: Knowns and unknowns. Acta Pharm Sin B.

[2] Ayoub SS (2021). Paracetamol (acetaminophen): A familiar drug with an unexplained mechanism of action. Temperature.

[3] Jaeschke H, Akakpo JY, Umbaugh DS, Ramachandran A (2020). Novel therapeutic approaches against acetaminophen-induced liver injury and acute liver failure. Toxicol Sci.

[4] Lv L, Xu C, Mo X, Sun HY, Bi H (2020). Green tea polyphenols protect against acetaminophen-induced liver injury by regulating the drug metabolizing enzymes and transporters. Evid Based Complement Alternat Med.

[5] Chiew AL, Isbister GK, Stathakis P, Isoardi KZ, Page C, Ress K (2023). Acetaminophen metabolites on presentation following an acute acetaminophen overdose (ATOM‐7). Clin Pharmacol Ther.

[6] McGill MR, Hinson JA (2020). The development and hepatotoxicity of acetaminophen: Reviewing over a century of progress. Drug Metab Rev.

[7] Jeong TB, Kim JH, Kim SH, Lee S, Son SW, Lim Y (2019). Comparison of toxic responses to acetaminophen challenge in icr mice originating from different sources. Lab Anim Res.

[8] Shi S, Wang L, Van der Laan LJ, Pan Q, Verstegen MM (2021). Mitochondrial dysfunction and oxidative stress in liver transplantation and underlying diseases: new insights and therapeutics. Transplantation.

[9] Niederreiter L, Tilg H (2018). Cytokines and fatty liver diseases. Liver Res.

[10] Win S, Than TA, Zhang J, Oo C, Min RWM, Kaplowitz N (2018). New insights into the role and mechanism of c‐Jun‐N‐terminal kinase signaling in the pathobiology of liver diseases. Hepatology.

[11] Huang K, Mukherjee S, DesMarais V, Albanese JM, Rafti E, Draghi IIA (2018). Targeting the PXR–TLR4 signaling pathway to reduce intestinal inflammation in an experimental model of necrotizing enterocolitis. Pediatr Res.

[12] Shojaie L, Iorga A, Dara L (2020). Cell death in liver diseases: A review. Int J Mol Sci.

[13] Effenberger M, Grander C, Grabherr F, Griesmacher A, Ploner T, Hartig F (2021). Systemic inflammation as fuel for acute liver injury in COVID-19. Dig Liver Dis.

[14] El Shaffei I, Abdel-Latif GA, Farag DB, Schaalan M, Salama RM (2021). Ameliorative effect of betanin on experimental cisplatin-induced liver injury; the novel impact of miRNA-34a on the SIRT1/PGC-1α signaling pathway. J Biochem Mol Toxicol.

[15] Latief U, Ahmad R (2018). Herbal remedies for liver fibrosis: A review on the mode of action of fifty herbs. J Tradit Complement Med.

[16] Li X, Sun R, Liu R (2019). Natural products in licorice for the therapy of liver diseases: progress and future opportunities. Pharmacol Res.

[17] Pramono S, Arifah FH, Pribadi FH, Nugroho AE (2018). Hepatoprotective activity of Curcuma xanthorrhiza roxb paracetamol-induced liver damage in rats and correlation with their chemical compounds. TJPS.

[18] Devaraj S, Ismail S, Ramanathan S, Yam MF (2014). Investigation of antioxidant and hepatoprotective activity of standardized Curcuma xanthorrhiza rhizome in carbon tetrachloride-induced hepatic damaged rats. Sci World J.

[19] Sahoo A, Jena S, Ray A, Dash KT, Nayak S, Panda PC (2021). Chemical constituent analysis and antioxidant activity of leaf essential oil of Curcuma xanthorrhiza. J Essent Oil-Bear Plants.

[20] Laksmitawati DR, Pratami DK, Widowati W, Kusuma HSW, Wijayanti CR, Wahyuni CD ( 2022). Antioxidant Properties of Curcuma longa and Curcuma xanthorrhiza Rhizomes.

[21] Widowati W, Darsono L, Lucianus J, Setiabudi E, Obeng SS, Stefani S (2023). Butterfly pea flower (Clitoria ternatea L ) extract displayed antidiabetic effect through antioxidant, anti-inflammatory, lower hepatic GSK-3β, and pancreatic glycogen on Diabetes Mellitus and dyslipidemia rat. J King Saud Univ Sci.

[22] Ginting CN, Lister INE, Girsang E, Widowati W, Yusepany DT, Azizah AM (2021). Hepatotoxicity prevention in acetaminophen-induced HepG2 cells by red betel (Piper crocatum Ruiz and Pav) extract from indonesia via antioxidant, anti-inflammatory, and anti-necrotic. Heliyon.

[23] Widowati W, Jasaputra DK, Gunawan KY, Kusuma HSW, Arumwardana S, Wahyuni CD (2021). Turmeric extract potential inhibit inflammatory marker in lps-stimulated marcophage cells. Int J Appl Pharm.

[24] Lister INE, Ginting CN, Girsang E, Nataya ED, Azizah AM, Widowati W (2020). Hepatoprotective properties of red betel (Piper crocatum Ruiz and Pav) leaves extract towards H2O2-induced HepG2 cells via anti-inflammatory, antinecrotic, antioxidant potency. Saudi Pharm J.

[25] Novilla A, Djamhuri DS, Nurhayati B, Rihibiha DD, Afifah E, Widowati W (2017). Anti-inflammatory properties of oolong tea (Camellia sinensis) ethanol extract and epigallocatechin gallate in LPS-induced RAW 264 7 cells. Asian Pac J Trop Biomed.

[26] Girsang E, Lister INE, Ginting CN, Nasution SL, Suhartina S, Munshy UZ (2020). Antioxidant and anti-inflammatory activity of Salacca zalacca (Gaertn ) voss peel ethanolic extract on lead induced fibroblast cells. Science and Technology Publication.

[27] Widowati W, Prahastuti S, Hidayat M, Hasianna ST, Wahyudianingsih R, Eltania TF (2022). Detam 1 black soybean against cisplatin-induced acute ren failure on rat model via antioxidant, antiinflammatory and antiapoptosis potential. J Tradit Complement.

[28] Hassanzadeh K, Vahabzadeh Z, Bucarello L, Dragotto J, Corbo M, Maccarone R (2022). Protective effect of curcuma extract in an ex vivo model of retinal degeneration via antioxidant activity and targeting the SUMOylation. Oxid Med Cell Longev.

[29] Lister INE, Ginting CN, Girsang E, Amansyah A, Chiuman L, Yanti NLWE (2019). Hepatoprotective effect of eugenol on acetaminophen-induced hepatotoxicity in HepG2 cells. J Phys Conf Ser.

[30] Girsang E, Ginting CN, Lister INE, Gunawan K, Widowati W (2021). Anti-inflammatory and antiaging properties of chlorogenic acid on UV-induced fibroblast cell. PeerJ.

[31] Rahmat E, Lee J, Kang Y (2021). Javanese turmeric (Curcuma xanthorrhiza Roxb ): Ethnobotany, phytochemistry, biotechnology, and pharmacological activities. Based Complement Alternat Med 2.

[32] Masfufatun M, Sari M, Jamilah A (2020). The antioxidant and hepatoprotective potential of temulawak (Curcuma xanthorrhiza Roxb) ethanol extract in paracetamol-induced rats. ICoSTE.

[33] Hasan AEZ, Falah S, Handharyani E, Dwicesaria MA (2023). Transaminase enzyme activity and histopathology evaluation of rat’s liver induced by DMBA with temulawak extract (Curcuma xanthorrhiza). J Adv Sci Eng Inf Techno.

[34] Ardiyanto D, Zulkarnain Z, Astana PRW, Triyono A, Novianto F, Fitriani U (2021). Efficacy of hepatoprotector jamu formula (combination of Curcuma longa, Curcuma xanthorrhiza, and Taraxacum officinale) compared to Fructus schizandrae extract in mild liver injury: A randomized controlled trial. IOP Conf Ser Earth Environ Sci.

[35] Lashgari NA, Khayatan D, Roudsari NM, Momtaz S, Dehpour AR, Abdolghaffari AH (2023). Therapeutic approaches for cholestatic liver diseases: The role of nitric oxide pathway. Naunyn Schmiedebergs Arch Pharmacol.

[36] Klein R, Nagy O, Tóthová C, Chovanová F (2020). Clinical and diagnostic significance of lactate dehydrogenase and its isoenzymes in animals. Vet Med Int.

[37] Majeed HJ, Ismail PA, Hassan LM (2020). Evaluation of serum nitric oxide levels and some biochemical parameters in patients with liver cirrhosis. Am J Biomed Sci Res.

[38] Puteri AIS, Sandhika W, Hasanatuludhhiyah N (2020). Effect of Javanese turmeric (Curcuma xanthorrhiza) extract on hepatitis model of alcohol-induced mice. J Kedokteran Brawijaya.

[39] Kritas SK, Ronconi G, Conti P, Pandolfi F (2019). Interrelationship between inflammatory cytokines (IL-1, IL-6, IL-33, IL-37) and acquired immunity. J Biol Regul Homeost Agents.

[40] Tran HB, Chen SC, Chaung HC, Cheng TC (2019). Molecular cloning of IL-6, IL-10, IL-11, IFN-ɤ and modulation of pro-and anti-inflammatory cytokines in cobia (Rachycentron canadum) after Photobacterium damselae subsp. Piscicida infection. Comp Biochem Physiol B Biochem Mol Biol.

[41] Megha KB, Joseph X, Akhil V, Mohanan PV (2021). Cascade of immune mechanism and consequences of inflammatory disorders. Phytomedicine.

[42] Ahmed H, Fazal N, Ahmad M R, Ijaz B, Bilal A Z, Ilyas S (2022). S-Allyl-L-cysteine-induced anti-inflammatory and anti-apoptotic effects in chondrocytes is associated with suppression of the mitochondrial inflammation pathway. Biol Clin Sci Res.

[43] Chen J, Ye C, Wan C, Li G, Peng L, Peng Y (2021). The roles of c-Jun N-terminal kinase (JNK) in infectious diseases. Int J Mol Sci.

[44] Farzaei MH, Zobeiri M, Parvizi F, El-Senduny FF, Marmouzi I, Coy-Barrera E (2018). Curcumin in liver diseases: A systematic review of the cellular mechanisms of oxidative stress and clinical perspective. Nutrients.

